# BrainCAT - a tool for automated and combined functional magnetic resonance imaging and diffusion tensor imaging brain connectivity analysis

**DOI:** 10.3389/fnhum.2013.00794

**Published:** 2013-11-21

**Authors:** Paulo Marques, José M. Soares, Victor Alves, Nuno Sousa

**Affiliations:** ^1^Life and Health Sciences Research Institute, School of Health Sciences, University of MinhoBraga, Portugal; ^2^ICVS/3B’s – PT Government Associate LaboratoryBraga/Guimarães, Portugal; ^3^Department of Informatics, University of MinhoBraga, Portugal

**Keywords:** brainCAT, fMRI, DTI, independent component analysis, tractography, connectivity, automated pipeline, multimodal neuroimaging

## Abstract

Multimodal neuroimaging studies have recently become a trend in the neuroimaging field and are certainly a standard for the future. Brain connectivity studies combining functional activation patterns using resting-state or task-related functional magnetic resonance imaging (fMRI) and diffusion tensor imaging (DTI) tractography have growing popularity. However, there is a scarcity of solutions to perform optimized, intuitive, and consistent multimodal fMRI/DTI studies. Here we propose a new tool, brain connectivity analysis tool (BrainCAT), for an automated and standard multimodal analysis of combined fMRI/DTI data, using freely available tools. With a friendly graphical user interface, BrainCAT aims to make data processing easier and faster, implementing a fully automated data processing pipeline and minimizing the need for user intervention, which hopefully will expand the use of combined fMRI/DTI studies. Its validity was tested in an aging study of the default mode network (DMN) white matter connectivity. The results evidenced the cingulum bundle as the structural connector of the precuneus/posterior cingulate cortex and the medial frontal cortex, regions of the DMN. Moreover, mean fractional anisotropy (FA) values along the cingulum extracted with BrainCAT showed a strong correlation with FA values from the manual selection of the same bundle. Taken together, these results provide evidence that BrainCAT is suitable for these analyses.

## INTRODUCTION

Brain connectivity studies have become popular nowadays with the combination of functional activation patterns using resting-state or task-related functional magnetic resonance imaging (fMRI) and diffusion tensor imaging (DTI) principles. While the brain is “at rest,” a large number of separated regions evidence statistical dependencies in the patterns of neuronal activity, which is assumed to be a measure of functional connectivity between regions, triggering what are commonly known as resting-state networks (RSNs; Biswal et al., 1995; Gusnard et al., 2001). RSNs (associated with language, vision, attention, executive processing, and other domains) are characterized by its consistency over time and across subjects (Damoiseaux et al., 2006; Fox and Raichle, 2007). These regions display high level of functional connectivity, pointing to the existence of structural pathways and connections (anatomical white matter tracts) that may facilitate the constant neuronal communication. Although still with some skepticism, there is an increasing consensus that changes in this intrinsic spontaneous brain activity under rest conditions can thus reflect functional, physiological, or structural alterations due to disease conditions (Buckner and Vincent, 2007; Arbabshirani et al., 2012; Brier et al., 2012; Shumskaya et al., 2012). Several studies have investigated this relationship between RSNs, such as default mode network (DMN), attention, visual and motor networks, and direct anatomical pathways architecture using DTI tractography (van den Heuvel et al., 2008, 2009; Greicius et al., 2009; Ystad et al., 2011; Cabral et al., 2012).

With constant technological and methodological advances, data analysis in neuroimaging has become totally dependent on computer-based processing, analysis, and result interpretation with complex workflows, run on increasingly larger datasets (Strother, 2006; Soares et al., 2013). In both fMRI and DTI studies, a common data processing pipeline engages several separated consecutive steps such as file format conversions, preprocessing, data processing, statistical analysis, and visualization of results (Haller and Bartsch, 2009; Hasan et al., 2011). Performing combined fMRI/DTI studies requires considerable workload and raises several problems. The generation of a huge amount of intermediate data can be hard to organize and handle; the definition of the correct step sequence might not be standardized; several processing steps require manual intervention or definition of several inputs which usually lead to very time consuming analysis, increasing the possibility of involuntary mistakes; different analysis could be performed in different coordinate systems (i.e., standard vs. native space). Some general sophisticated pipeline environments have been developed to automate and simplify neuroimaging analysis [e.g., LONI Pipeline Processing Environment (Rex et al., 2003; Patel et al., 2010), NA-MIC Kit (Pieper et al., 2006), and FisWidgets (Fissell et al., 2003)]. However, in these cases, users need to specify their own pipeline using the modules made available by the environment, requiring deep neuroimaging knowledge and technical expertise about each module. Despite the availability of several free or commercial tool packages for DTI or fMRI data analysis, there is a lack of solutions for an optimized multimodal analysis combining these techniques.

These issues contribute to difficulties in reproducing analyses, diminishing their sensitivity and accuracy and reducing the appeal of combined fMRI/DTI analysis. In order to overcome these issues, newcomers to the neuroimaging field need a huge learning investment. As such, researchers would benefit from an automated and simplified tool to help in the processing and analysis of combined fMRI/DTI data. To overcome this gap, we developed a new tool, entitled brain connectivity analysis tool (BrainCAT), for an intuitive multimodal fMRI/DTI analysis using a friendly graphical user interface (GUI) that encloses part of the knowledge about the whole process. BrainCAT ultimately enables the user to go from raw fMRI and diffusion weighted imaging (DWI) data to results without the need to know how to manually perform image-processing operations and with minimal user intervention. Starting with raw Digital Imaging and Communications in Medicine (DICOM) images properly arranged in a directory tree, BrainCAT implements a predefined pipeline for fMRI and DWI data preprocessing, independent component analysis (ICA) of the fMRI data and combination with DTI tractography analysis.

The provision of such tool would allow the controlled, optimized, and automated processing of large datasets in a reasonable amount of time and with minimal user intervention. In order to validate BrainCAT, it was used to test for the effect of age in the DMN white matter connectivity of an elderly population. Based on previous literature, we expected to find white matter tracts interconnecting the precuneus/posterior cingulate cortex (PCC) and the medial frontal cortex (MFC) and between the bilateral medial temporal lobe (MTL) clusters and the PCC cluster (Greicius et al., 2009; van den Heuvel et al., 2009). Moreover, no significant correlation between fractional anisotropy (FA) and age is expected along the cingulum bundle (Michielse et al., 2010; Sullivan et al., 2010). In order to provide some comparative results, the FA profiles along cingulum were also extracted with more traditional methods and the results were compared with the ones obtained with BrainCAT.

## MATERIALS AND METHODS

### BrainCAT

Brain connectivity analysis tool was developed as a Macintosh Operating System (Mac OS) application, written in Objective-C using the Cocoa framework and follows the model-view-controller (MVC) design pattern. X-Code was used as the programming environment. The interface was designed with interface builder and it was compiled with the GCC 4.2 compiler under a Mac OS X 10.6.8.

**FIGURE 1:**
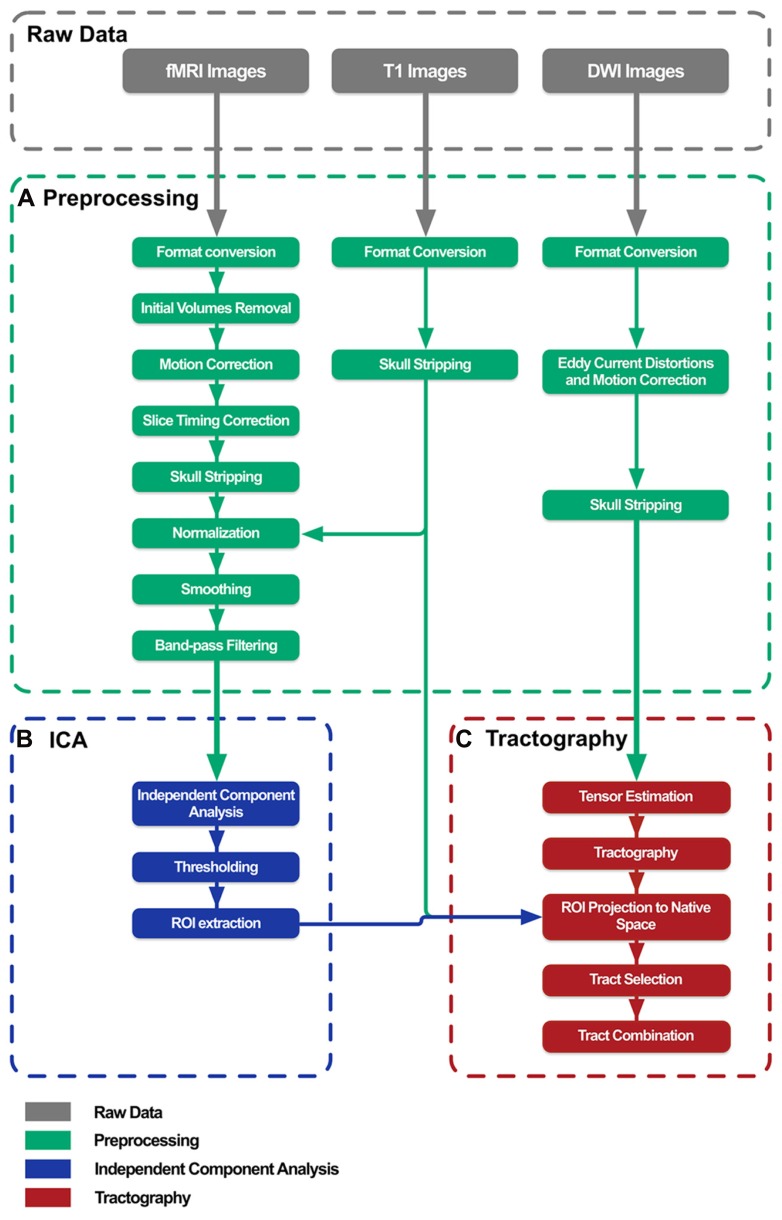
**BrainCAT data processing pipeline overview.** The pipeline was defined in order to preprocess raw data **(A)**, perform independent component analysis on fMRI data **(B)**, and combine its results with white matter tracts reconstructed with DTI tractography **(C)**.

Brain connectivity analysis tool was developed as an application with a simple goal: to combine multiple freely available tools in order to implement an optimized and automated data processing pipeline to combine ICA results with tractography outcomes. BrainCAT pipeline definition (**Figure 1**) was based on previous published multimodal fMRI/DTI studies (van den Heuvel et al., 2008, 2009; Greicius et al., 2009). The steps included are the ones recommended to perform typical analysis, even though the user has the option to exclude them from the pipeline, and personalize each step according to the specific analysis.

**FIGURE 2:**
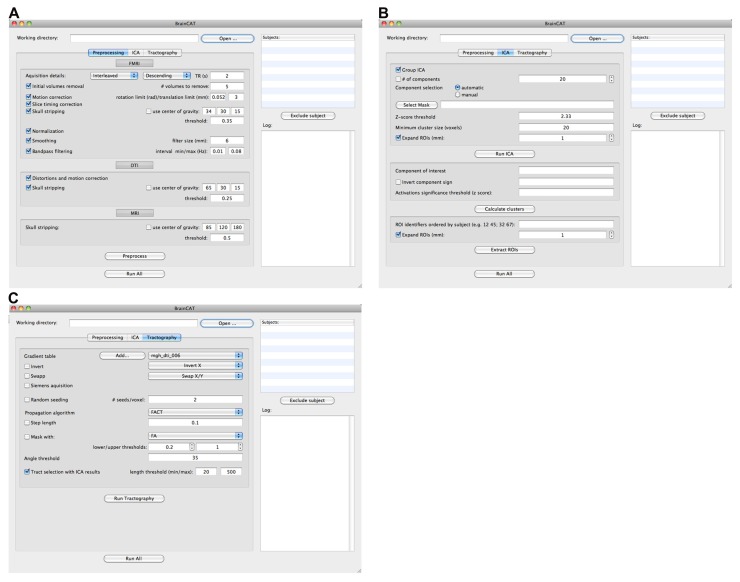
**BrainCAT’s user interface.** It is organized in three different tabs reflecting different functionalities: preprocessing **(A)**, ICA analysis **(B)**, and DTI tractography **(C)**.

Briefly, BrainCAT organized such that the user is able to (i) preprocess the data of both acquisitions (**Figures 1A and 2A**), (ii) run ICA on the fMRI data and extract regions of interest (ROIs) from the results (**Figures 1B and 2B**), (iii) run tractography on the DTI data and combine it with the ROIs from the ICA analysis (**Figures 1C and 2C**). As so, it presents three different tabs for each of these sections (**Figure 2**). To implement these functions, BrainCAT uses a set of freely available software tools distributed with MRICron (Rorden and Brett, 2000), FSL (Smith et al., 2004), and TrackVis (Wedeen et al., 2008). The main development considerations and functionalities will be described below.

#### Preparing the data for analysis

Neuroimaging studies usually consist of large datasets composed by hundreds or thousands of images, from several acquisitions performed on each participant. Most software tools require the user to manually input each subject and acquisition, one at a time.

To overcome this limitation, BrainCAT makes use of the organization “Study – Subject – Acquisition – Images” to simplify the input of the data. The user only needs to organize the study’s data in a working directory containing one folder by subject. Each subject’s folder has three mandatory directories for the fMRI, DTI, and T1 structural MRI acquisitions containing the images in DICOM format, named FMRIDCM, DTIDCM, and MRIDCM, respectively. Alternatively, the images can also be inputted in NIfTI format and should be placed under properly named directories (i.e., FMRI, DTI, and MRI, respectively). If the data is organized accordingly, the user only needs to input the working directory of the study and BrainCAT automatically detects the subjects included in the study, creates a new study and enables the user to start the workflow.

#### Preprocessing

With BrainCAT, all the preprocessing steps (**Figure 1A**) for fMRI and DTI data are performed at once after pressing one single button.

When starting the preprocessing stage, BrainCAT searches for the subjects’ data in NIfTI format. If some data is missing in all or some subjects, BrainCAT will search for the DICOM files and, if found, it will convert those files into NIfTI file format calling *dcm2nii* tool. This tool is widely used for this purpose and it’s distributed along with MRICron.

For fMRI preprocessing, the first step performed by BrainCAT consists in removing the initial volumes of the acquisition so that the instability of the main magnetic field at the beginning of the acquisition does not interfere with the results. For this, a tool called *fslroi*, part of the FSL software package, is used.

The removal of initial volumes is followed by motion correction to compensate for involuntary head movement, so that each anatomical landmark stays in the same position across all the volumes of the acquisition. BrainCAT corrects this motion with a rigid alignment of all the volumes to the mean of the functional acquisition. FSL’s *mcflirt* tool is used for this purpose. It also outputs charts of the extent of translational and rotational movement and excludes from the study the subjects whose head movement exceeds the thresholds defined by the user.

During the acquisition, each slice is acquired in a different time-point. The fMRI analysis assumes that an entire volume is acquired at the same time meaning that timing differences between slices should be corrected. In BrainCAT, this adjustment is made using the slice acquired in the middle of repetition time (TR) as reference and calling another FSL tool, named *slicetimer*. The use of the mean reduces the amount of interpolation needed since timing differences should be reduced. In sequential acquisitions the steps order is inverted, thus motion correction is performed before slice timing. Although there is no consensus regarding the order of these two steps, this was found to be the most common approach (Chen et al., 2009; Sladky et al., 2011); BrainCAT carries out this order inversion automatically.

In fMRI studies, the analysis is limited to brain structures. As so, BrainCAT performs skull stripping in a two-step procedure. In the first step, a brain mask is calculated from the mean volume of the functional scan, using FSL’s *bet* program. Then, *fslmaths* command-line tool from FSL enables BrainCAT to apply the mask to all volumes in the acquisition, removing the non-brain structures. This step is crucial for the subsequent normalization step. Importantly, BrainCAT enables the user to adjust the main parameters that drive the skull-stripping procedure, the center of gravity of the brain and the fractional intensity threshold.

To perform group analysis, all subjects’ data must be in a common standard space and the acquisitions need to be spatially transformed so that each anatomical landmark is in the same position across all the subjects (Evans et al., 2012). In BrainCAT, the normalization step comprises three different stages. First, a rigid alignment of the mean functional acquisition to the structural scan is calculated. Afterwards, the structural volume is affine registered to the Montreal Neurological Institute (MNI) standard space. Finally, both transformations are concatenated and the resulting transformation matrix is applied to the functional scans. The transformations are estimated and applied with FSL’s *flirt* tool with interpolation to 2 mm isotropic voxel size. At the end, BrainCAT outputs 2D images with some orthogonal views of the normalized brain that enable the user to check the accuracy of the normalization. It also automatically applies skull stripping to the structural acquisition, which is crucial to obtain a good alignment. As in the fMRI skull-stripping step, the user can define the center of gravity of the brain and the fractional intensity threshold to optimize the procedure.

In order to increase signal-to-noise ratio (SNR) and reduce residual inter-subject misregistrations, the user can set BrainCAT to apply spatial smoothing to the functional volumes. This is done by the convolution of the images with a tridimensional Gaussianfunction, meaning that the resulting voxels will represent a weighted mean of itself and the neighboring voxels. BrainCAT allows the user to specify the full width at half maximum (FWHM) to control the extent of smoothing which is applied with *fslmaths*.

The final step of the fMRI preprocessing pipeline consists in the application of a band-pass filter to the time-series. RSNs have been associated to low frequency (0.01–0.08 Hz) BOLD fluctuations. BrainCAT performs this step combining a high-pass filter with a low-pass filter. This is performed with another call to *fslmaths*. The user has also the option to skip one of the filters since there is growing evidence that the higher frequency range can still contain important neuronal driven fluctuations (Feinberg et al., 2010).

After the fMRI preprocessing, BrainCAT starts the DTI preprocessing stage. As for fMRI scans, head movement has also drastic effects on DWI acquisitions. Furthermore, the DWI acquisitions are susceptible to eddy currents caused by the gradients applied. These currents produce distortions to the images that should be corrected (Le Bihan et al., 2006). BrainCAT corrects these distortions using *eddy_correct* script distributed with FSL. This script invokes the *flirt* program to affine align all of the DWI volumes to the b0 volume since this volume is not affected by eddy current distortions. Motion correction also consists in the alignment of the volumes to a reference volume, which means that both corrections are performed at once. Furthermore, BrainCAT uses another FSL’s script, called *fdt_rotate_bvecs*, to rotate the gradient vectors information accordingly to the spatial transformations applied to their corresponding volumes.

Diffusion tensor imaging acquisitions should also be skull stripped to limit the analysis to the brain. This step is performed in a similar manner to the skull-stripping process of the functional scan, calling *bet*, with the difference that the reference volume is the b0 volume instead of the functional mean.

#### Independent component analysis

After preprocessing the data, the user can start the fMRI analysis (**Figure 1B**). BrainCAT was developed to allow the extraction of ROIs from the ICA results of the fMRI data. This can be done in a fully automated way or in a semi-automated approach.

In automatic mode, BrainCAT runs ICA, invoking the command line version of MELODIC, the well-known ICA tool from FSL (Beckmann and Smith, 2004). BrainCAT enables the user to perform group ICA using the concatenation approach for decomposition of MELODIC in order to search for common spatial patterns among subjects. The user can set the number of components to extract or let MELODIC estimate it. After the component estimation, BrainCAT also calls *dual_regression* program in order to retrieve each subject’s version of the group components. This enables the user to further use these estimates to perform other analyses outside the scope of BrainCAT such as group comparisons or correlation analyses using tools like *randomize*. After performing group ICA, BrainCAT selects the component of interest from which to extract ROIs based on its similarities to the mask specified by the user. It computes cross-correlations between the mask and every independent component map, using *fslcc* program from FSL, selects the one with the highest cross-correlation, extracts it and inverts its sign if necessary. Finally, the *clusters* tool from FSL is used to isolate the clusters that survive to a predefined *Z*-score threshold and every cluster that survives to the cluster size threshold is separated into one NIfTI file using *fslmaths*.

The semi-automated approach enables the user to select the independent component to be used based on visual inspection, instead of using a RSN mask. For this purpose BrainCAT calls *fslview*, a viewer distributed with FSL, and loads the 4D dataset containing all the estimated components overlaid on the MNI152 T1 template. The ROI selection will also be based on visual inspection. After isolating the clusters, BrainCAT calls *fslview* again with the resulting clusters overlaid on the MNI152 T1 template. The user can easily identify the different clusters by their intensity value. Then it allows the user to specify the ROIs to be extracted by inputting the corresponding intensity value and separates them in different NIfTI files.

Alternative to the group components estimation, BrainCAT also enables the user to estimate the individual components of each subject directly, to be used for instance in group comparisons.

Finally, BrainCAT outputs for each cluster, the results of the ICA analysis: clusters size in number of voxels, regional peaks of *Z*-score, their MNI coordinates, and the corresponding brain region according to the Harvard–Oxford Cortical Structural Atlas (Flitney et al., 2007).

#### DTI tractography

For the tractography (**Figure 1C**), BrainCAT works similar to Diffusion Toolkit. Using the preprocessed DWI images, it calls *dti_recon* command line program to fit the tensors and estimate the scalar metrics. Then the *dti_tracker* program, also distributed with Diffusion Toolkit is called in order to run the tractography. Several parameters that drive the tracking procedure can be customized. For the seeding stage, the user can opt to use random seeding and set the total of seeds placed in each voxel, instead of using a single seed centered in each voxel. For the tracking phase, the user can choose which fiber tracking algorithm to use and the step length between iterations. For the termination stage, the user can define the angle threshold and scalar thresholds (e.g., FA). Finally, the *spline_filter* tool is used in order to provide smoother tracks and reduce the amount of disk space required.

In order to combine the ROIs from the ICA analysis with the tractography of each subject, BrainCAT transforms them to the native space of the DWI acquisition. This step is performed similar to the normalization of the functional images, with the inverse transformation of the normalization being applied to each ROI. TrackVis command line version is then called in order to filter the tracts that link each possible pairs of ROIs. Several statistics are extracted for the resulting tract: mean track length, number of tracks, number of voxels, volume, mean FA, and mean diffusivity (MD).

With the isolated tracts from each individual, BrainCAT transforms them into MNI standard space, calling *track_transform* program from Diffusion Toolkit and combines the results in one single tract using *track_merge* tool, also distributed with Diffusion Toolkit. This tract represents the sum of the results obtained for each subject.

### THE EFFECT OF AGING IN THE STRUCTURAL CONNECTIVITY OF THE DEFAULT MODE NETWORK OF THE ELDERLY

To better illustrate its capabilities we will present some experimental results obtained using BrainCAT in a combined fMRI/DTI analysis of the effects of aging in the DMN structural connectivity.

#### Participants

A total of 106 volunteer subjects matched for gender (53 males, 53 females) with mean age of 65.41 ± 8.31 years participated in this study. The current study was part of the SWITCHBOX project whose goals and tests were explained to all participants and all gave informed written consent. The study was conducted in accordance with the principles expressed in the Declaration of Helsinki and was approved by the Ethics Committee of Hospital de Braga (Portugal).

#### Image acquisition

Subjects were scanned on a clinical approved Siemens Magnetom Avanto 1.5 T (Siemens Medical Solutions, Erlangen, Germany) at Hospital de Braga using a Siemens 12-channel receive-only head coil.

All the subjects underwent a task free functional scan with gradient echo T2* weighted echo-planar images (EPIs) acquired with the following parameters: 180 volumes; TR = 2000 ms; echo time (TE) = 30 ms; flip angle = 90°; in-plane resolution = 3.5 × 3.5 mm^2^; 30 interleaved slices; slice thickness = 4.5 mm; field of view (FoV) = 224 mm; imaging matrix 64 × 64. During the functional acquisition, participants were instructed to keep the eyes closed and to think in nothing particular.

The DWI were acquired using spin-echo echo-planar imaging (SE-EPI), with maximum gradient amplitudes of 30 mT/m allowed by the equipment. Imaging parameters were: TR = 8.8 s; TE = 99 ms; 61 interleaved axial slices with voxel size 2 × 2 × 2 mm^3^; FoV = 240 mm; imaging matrix 120 × 120; providing continuous whole brain coverage. Following an acquisition without diffusion sensitization with *b* = 0 s mm^-^^2^, DWI were acquired with diffusion gradients applied with *b* = 1000 s mm^-^^2^, along 30 non-collinear directions. The sequence was repeated in two successive runs in order to increase the SNR of the parametric maps to be computed.

A structural 3D magnetization prepared rapid gradient echo (MPRAGE) scan was also acquired with 176 sagittal slices, TR = 2730 ms, TE = 3.48 ms, flip angle = 7°, in-plane resolution = 1 × 1 mm^2^ and slice thickness = 1 mm.

#### Data processing

For the data preprocessing and analysis, the standard and automated pipeline was employed. This means that the fMRI data preprocessing included the removal of the first five volumes of the acquisition, motion correction, slice timing correction, skull stripping, spatial normalization, smoothing with a 8 mm FWHM kernel, and band-pass temporal filtering (0.01–0.08 Hz). The DWI images underwent motion and distortions correction and skull stripping as preprocessing steps.

After preprocessing, the fMRI images underwent group ICA with automatic estimation of the number of independent components. For the component selection, the mask of the dorsal DMN provided by the Functional Imaging in Neuropsychiatric Disorders Lab (Shirer et al., 2012) was used as reference. A combined threshold of *Z*-score > 3.09 (*p* < 0.001) and cluster size > 60 voxels was used for ROI extraction. We found this threshold to provide a good tradeoff between statistical significance and sufficient cluster size for the combination with tractography results.

For the tractography analysis, tensor estimation was performed using the standard Siemens 30 directions gradient table. The “Interpolated Streamline” algorithm was then used to reconstruct the tracks. Five seeds were randomly placed in each voxel and fiber tracking was stopped when voxels with FA values lower than 0.2 were reached or when the angle change was greater than 35°. In order to filter the reconstructed tracts linking the DMN clusters, the option to combine tractography results with ICA was enabled.

#### Statistical analysis

A statistical analysis was performed in order to test the effect of aging in the white matter tracts linking the functional clusters that compose the DMN. For each subject, mean FA and mean MD of each track outputted by BrainCAT were used in a separate correlation analyses against age. The results were considered significant at *p* < 0.05.

#### Comparative analysis

In order to provide some comparative results against more traditional methods, we also performed a correlation analysis of the FA profiles along the cingulum extracted using BrainCAT with the profiles obtained with the manual selection of cingulum bundle. In order to do this, for each subject, two ROIs were drawn just above the corpus callosum, one on the most anterior part and another on the most posterior part. These ROIs were drawn overlaid on the FA maps of each subject and we visually confirmed that both were crossed by the cingulum. The tract selection procedures were performed with TrackVis. Only the tracts that trespassed both ROIs were considered for the extraction of the mean FA along the cingulum. Two correlational analysis were performed: one with age and another with the mean FA values of the tracts interconnecting the PCC and MFC clusters of the DMN, extracted with BrainCAT. The results were considered significant at *p* < 0.05.

## RESULTS

### BrainCAT: BRAIN CONNECTIVITY ANALYSIS TOOL

Developed as a Mac OS X application, BrainCAT implements an automated data processing pipeline for combined analysis of resting state fMRI and DTI data. This pipeline includes the most important data processing procedures necessary to perform such analysis. In order to implement each data processing step, only freely available and validated tools were used, namely the ones distributed with MRICron, FSL, Diffusion Toolkit, and TrackVis. As such, in order to use BrainCAT, users only need to have previously installed these software packages. This new application can be freely downloaded at .

Besides enabling the data preprocessing of both MRI acquisitions, the ICA analysis and the combination of these results with DTI tractography, BrainCAT also implements some other small useful features. As an example, every command call is written to a log file so that the user can check the commands run and the parameters used for each command. Besides this, BrainCAT arranges the resulting files in folders (e.g., one for the results of the preprocessing of each acquisition, one for the ICA analysis, and one for the tractography results) and names each file with a set of predefined codes that facilitate the identification of each file. **Tables 1** and **2** summarize, respectively, the main files and folders produced by BrainCAT and their corresponding contents.

**Table 1:** Files produced by BrainCAT.

Filename	Contents
{SUBJID}_diff.nii.gz	Original DWI in NIfTI format
{SUBJID}_diff.bval	Diffusion *b*-values text file
{SUBJID}_diff.bvec	Diffusion gradients’ directions text file
{SUBJID}_diff.nii.gz	Original DWI in NIfTI format
{SUBJID}_diff_b0.nii.gz	b0 image of the diffusion acquisition
{SUBJID}... _eddy.nii.gz	Diffusion images after eddy current and motion correction
{SUBJID}... _eddy.ecclog	eddy_correct log file with the transformations applied during eddy current correction
{SUBJID}_fnc.nii.gz	Original functional images in NIfTI format
{SUBJID}_... _vol.nii.gz	Functional images after removal of initial volumes
{SUBJID}_... _stime.nii.gz	Functional images after slice timing
{SUBJID}_... _mean.nii.gz	Mean functional image
{SUBJID}_... _mcf.nii.gz	Functional images after motion correction
{SUBJID}... _mcf.par	Motion correction estimated parameters for translation and rotation
{SUBJID}... _rot.png	Chart image with motion parameters for rotations
{SUBJID}... _trans.png	Chart image with motion parameters for translations
{SUBJID}_... _smooth.nii.gz	Functional images after smoothing
{SUBJID}_... _filter.nii.gz	Functional images after filtering
{SUBJID}_str.nii.gz	Original structural images in NIfTI format
{SUBJID}_... _bet.nii.gz	Images after skull stripping
{SUBJID}_... _bet_mask.nii.gz	Skull stripping brain mask
{SUBJID}_... _mni.nii.gz	Normalized images
{SUBJID}_... _2str.mat	Affine transformation matrix to native structural space
{SUBJID}_... _2mni.mat	Affine transformation matrix to MNI standard space
{SUBJID}_... _2nat.mat	Affine transformation matrix from MNI standard space to native space
melodic_IC.nii.gz	4D Melodic output file with the ICA results
component_{compID}.nii.gz	Independent component extracted to be analyzed. compID is the compIDth image from the melodic_IC.nii.gz file
component_{compID}_cls.nii.gz	Image containing the clusters resulting from the thresholding of the compIDth independent component
component_{compID}_roi_{roiID}.nii.gz	Mask image of the cluster with roiID intensity
component_{compID}_clusterinfo.txt	Clusters data text file
component_{compID}_localmaxinfo.txt	Local maxima data text file
component_{compID}_atlasregions.txt	Local maxima
{SUBJID}_roi_{roi1ID}_roi_{roi2ID}.trk	Local maxima corresponding atlas regions
{SUBJID}_dwi.nii.gz	Diffusion images used for tensor estimation
{SUBJID}_tensor.nii.gz	Estimated tensor data
{SUBJID}_adc.nii.gz	MD scalar map
{SUBJID}_e1.nii.gz	First eigenvalue scalar map
{SUBJID}_e2nii.gz	Second eigenvalue scalar map
{SUBJID}_e3.nii.gz	Third eigenvalue scalar map
{SUBJID}_v1.nii.gz	First eigenvector data
{SUBJID}_v2.nii.gz	Second eigenvector data
{SUBJID}_v3.nii.gz	Third eigenvector data
{SUBJID}_fa.nii.gz	FA scalar map
{SUBJID}_fa_color.nii.gz	Colored FA scalar map
{SUBJID}.trk	Whole brain tractography file
{SUBJID}_roi_{roi1ID}_roi_{roi2ID}.trk	Track file containing the tracks that connect ROIs roi1ID and roi2ID
{SUBJID}_roi_{roi1ID}_roi_{roi2ID}_vol.nii	NIfTI image representative of the tracks that connect ROIs roi1ID and roi2ID
{SUBJID}_roi_{roi1ID}_roi_{roi2ID}_mni.trk	Track file transformed into MNI standard space
{SUBJID}_tractresults.txt	Tractography results text file
grouptrack_roi_{roi1ID}_roi_{roi2ID}.trk	Cross-subject combined tracts connecting ROIs roi1ID and roi2ID

*Between braces words refer to the corresponding IDs (i.e., SUBJID, id of the subject being analyzed; compID, number of the independent component; roiID, roi1ID, and roi2ID: intensity value of the ROI). Ellipsis represents any combination of suffixes, depending on the workflow settings.*

**Table 2:** Folders produced by BrainCAT.

Folder	Contents
{SUBJID}/FMRI	Original NIfTI functional images and the images and files resulting from preprocessing of the functional acquisition of subject SUBJID
{SUBJID}/MRI	Original NIfTI structural images and the images and files resulting from preprocessing of the structural acquisition of subject SUBJID
{SUBJID}/DTI	Original NIfTI diffusion images and the images and files resulting from preprocessing of the diffusion acquisition of subject SUBJID
{SUBJID}/Tractography	Tensor fitting and tractography results of subject SUBJID
{SUBJID}/ICA/	Independent component analysis results in SUBJID native space
{SUBJID}/ICA/GroupICA_ROIS	ROI masks extracted from GroupICA analysis in the native space of diffusion acquisition
GroupICA	Results of the GroupICA analysis
GroupICA/Individual_ICs	Individual subject independent components resulting from dual_regression of the GroupICA results
GroupTracts	Combined cross-subject results of the tractography analysis

*{SUJID} represents the id of the subject being analyzed.*

Regarding the execution time necessary to analyze each dataset no real tests were performed since several aspects needed to be accounted for (e.g., acquisition, choice of parameters, and computer specifications). However, in order to provide some reference, the data processing of the dataset used to test BrainCAT’s validity took approximately 86 h on an iMac with a 3.06 GHz Intel Core 2 Duo processor and 8 GB of RAM.

### THE AGE EFFECT ON DMN’S UNDERLYING STRUCTURAL CONNECTIONS

**FIGURE 3:**
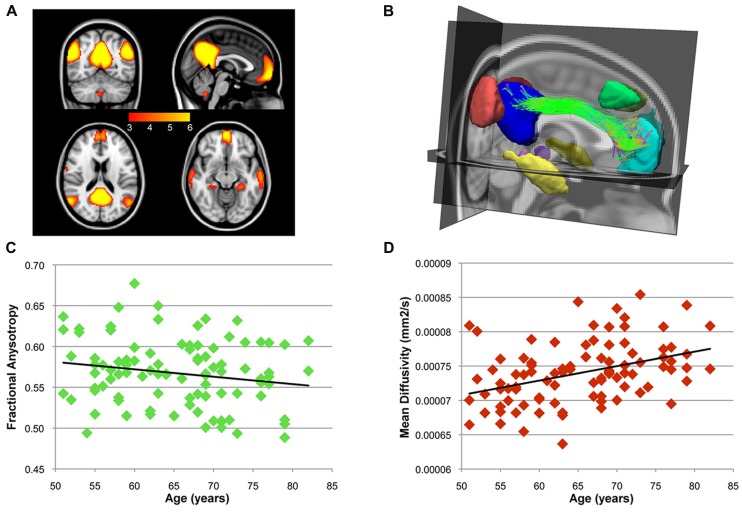
**After the group ICA, BrainCAT correctly identified the DMN.** **(A)** The DMN clusters that survived the peak threshold of *Z* > 3.09. The cingulum bundle was found to structurally interconnect the PCC and medial prefrontal cortex (MPF) clusters **(B)**. A significant negative correlation (*r* = -0.18; *p* = 0.079) was found between age and FA along the cingulum bundle **(C)** while correlation analysis with MD yielded a significant positive correlation **(D**; *r* = 0.39; *p* < 0.)

After ICA analysis, BrainCAT correctly identified the DMN component and, after peak and cluster thresholding, several clusters were extracted (**Figure 3A**). Among these, the PCC, MFC, and bilateral MTL and inferior temporal lobe (ITL) clusters, commonly reported as part of the DMN, survived the combined peak (*p* < 0.001) and extent thresholds (minimum cluster size of 60 voxels).

After combining these clusters with DTI tractography, almost every subject presented bilateral white matter fibers interconnecting the PCC and the MFC clusters forming the cingulum bundle (**Figure 3B**). Moreover, correlation analysis between age and FA and MD profiles along this tract yielded a non-significant negative correlation with FA (*r* = -0.18; *p* = 0.079 – **Figure 3C**) and a significant positive correlation with MD (*r* = 0.39; *p* < 0.0001 – **Figure 3D**). These patterns of functional and structural connectivity are in line with the expected results. The correlation analysis of the mean FA values along the cingulum between the more traditional approach and BrainCAT resulted in a strong positive correlation between both methods (*r* = 0.79; *p* < 1 × 10^-^^22^).

## DISCUSSION

As stated previously, BrainCAT is, to the best of our knowledge, the first tool designed to implement an automated and intuitive fMRI/DTI combined-processing pipeline. We tried to follow as possible the processing pipelines described in previous studies. Similar tools with automated pipeline implementations like the data processing assistant for resting-state fMRI (DPARSF; Chao-Gan and Yu-Feng, 2010) for fMRI data preprocessing and functional connectivity analysis and the Pipeline for Analyzing braiN Diffusion imAges (PANDA; Cui et al., 2013), which implements a fully automated pipeline for DWI data processing with some degree of parallelization, are freely available. The resemblances, however, are confined to the automated data processing; in fact, unlike BrainCAT, these tools are not tailored for multimodal analysis. There is an insufficient offer of optimized platforms for multimodal neuroimaging studies, accessible to the general user. Typical solutions are accessible in single software packages such as Freesurfer, Brainvoyager (Goebel, 2012), and FSL. However, there are intrinsic limitations in working only with one software and compatibility issues associated with other packages. Actually, these software packages do not allow combining fMRI ICA with tractography results. A possible solution for multi-package analysis is LONI Pipeline processing environment; however, we believe that it is not as intuitive as BrainCAT and it is necessary that the user defines and configures its own pipeline manually.

Brain connectivity analysis tool was also designed to have a very compact and simple GUI, and to reduce to the minimum the amount of parameters to be set; such strategy makes it easier to use. We find BrainCAT easier to use than the usual multimodal workflows, where users have to handle multiple software packages and file formats. It is also very flexible since it is able to perform both individual and group analyses; it can be used for multimodal or single modality studies of fMRI or DTI. Although no real comparative study has been done, we believe this new tool also reduces the amount of time necessary to run the data analysis. This can be explained by two reasons: on one hand by reducing the amount of parameters necessary to define, it reduces the amount of time necessary to start the data processing and on the other it executes as many processing steps as possible after a button is pressed. In this way, the user is free to do any other tasks while BrainCAT is processing the data, as no user intervention is necessary during this process. Another advantage is that it reduces significantly the number of user involuntary errors in processing steps, data manipulation and software handling. Besides all these benefits, we consider, especially for the newcomers, the fact that the user needs to manipulate one single application that implements a processing pipeline that enables the combination of results from fMRI and DTI analysis as the main benefit of using BrainCAT.

In order to test BrainCATs’ reliability, we analyzed a large dataset in a study of healthy aging. We focused on the white matter tracts interconnecting the typical DMN brain regions, especially the cingulum bundle since it has been widely described as the structural connector between the PCC and MFC clusters (van den Heuvel et al., 2008, 2009; Greicius et al., 2009). As expected, our results demonstrate that the cingulum bundle interconnects these two regions, but also reveal a non-significant reduction of FA in the cingulum with increasing age which is consistent with previously reported results (Sullivan et al., 2010). On the contrary, MD increased significantly with aging. An increase in diffusivity along life span has also been reported in this bundle, mainly for radial diffusivity (Davis et al., 2009) which has been associated to myelodegeneration. Importantly, herein, we show the strong positive correlation between FA estimates along the cingulum obtained with BrainCAT and with manual selection of the same bundle demonstrates that the methods employed in BrainCAT can be used to study the profiles of DTI scalar metrics along white matter tracts.

Our findings are concordant with the typical aging pattern of white matter microstructure that is characterized by a decrease in FA accompanied by an increase in diffusivity (Pfefferbaum et al., 2005). The magnitude of the increase in FA–MD ratio varies across brain regions but is always greater in elder subjects (Pfefferbaum and Sullivan, 2003). This altered ratio suggests that decreased brain white matter intravoxel coherence is attributable, at least in part, to the accumulation of interstitial or intracellular fluid, or both fluid compartments (Silva et al., 2002; Pfefferbaum et al., 2005) and may reflect age-related loosening of myelin, dense cytoplasm, and formation of fluid-filled balloons (Peters and Sethares, 2003). Importantly, several studies have correlated these age-related changes in the FA–MD ratio of white matter with cognitive performance both in healthy aged subjects (Grieve et al., 2007) and in neuropathological conditions (Head et al., 2004), thus reinforcing the relevance of such measurements for studies aiming to establish structural–functional relationships.

Brain connectivity analysis tool, however, also presents limitations. One of them relates to the fact that all preprocessing is done at once. If the results are not satisfactory the user may have to re-run most of the preprocessing with different parameters. The fact that the user does not need technical expertise to perform each of the processing steps can also be a limitation due to the fact that this could also lead to misinterpretations of the results.

A final note to highlight that we expect extensive interactions with other researchers/users to expand the functionalities available in BrainCAT. In upcoming versions, BrainCAT will keep being updated to include the most suitable and accurate processing steps available according to the literature. We intend to incorporate in BrainCAT new modules to perform other neuroimaging combined multimodal analysis, such as: automated volumetric/DTI analysis; other methodologies to access connectivity measures (e.g., seed-correlation analysis, probabilistic tractography); graph theory analysis. We also expect to develop some new features that will enable advanced users to further customize the pipeline. In summary, BrainCAT can simplify and potentiate combined fMRI/DTI studies in the near future and contribute to a better understanding of the brain connectivity that is the focus of ongoing neuroimaging research projects (e.g., Human Connectome Project and the Developing Human Connectome Project).

## Conflict of Interest Statement

The authors declare that the research was conducted in the absence of any commercial or financial relationships that could be construed as a potential conflict of interest.

## AUTHOR CONTRIBUTIONS

Paulo Marques and José M. Soares contributed in literature search, figures, study design, data collection, data analysis, data interpretation, and writing. Victor Alves and Nuno Sousa contributed in study design, data interpretation, and writing.
